# Forecasting virus outbreaks with social media data via neural ordinary differential equations

**DOI:** 10.1038/s41598-023-37118-9

**Published:** 2023-07-05

**Authors:** Matías Núñez, Nadia L. Barreiro, Rafael A. Barrio, Christopher Rackauckas

**Affiliations:** 1 Consejo Nacional de Investigaciones Científicas y Técnicas (CONICET), Buenos Aires, Argentina; 2 Departamento Materiales Nucleares, Centro Atómico Bariloche, Comisión Nacional de Energía Atómica (CNEA), Bariloche, Argentina; 3 Ecología cuantitativa, Instituto de Investigaciones en Biodiversidad y Medioambiente, Bariloche, Argentina; 4 Instituto de Investigaciones Científicas y Técnicas para la Defensa (CITEDEF), Buenos Aires, Argentina; 5 Instituto de Física, Universidad Nacional Autónoma de México, Apartado Postal 20-365, México, 04510 Mexico; 6 Computer Science & Artificial Intelligence Laboratory (CSAIL), Massachusetts Institute of Technology, Cambridge, MA 02142 USA; 7 JuliaHub Inc., Cambridge, MA USA; 8 Pumas-AI, Baltimore, MD USA

**Keywords:** Epidemiology, Machine learning

## Abstract

During the Covid-19 pandemic, real-time social media data could in principle be used as an early predictor of a new epidemic wave. This possibility is examined here by employing a neural ordinary differential equation (neural ODE) trained to forecast viral outbreaks in a specific geographic region. It learns from multivariate time series of signals derived from a novel set of large online polls regarding COVID-19 symptoms. Once trained, the neural ODE can capture the dynamics of interconnected local signals and effectively estimate the number of new infections up to two months in advance. In addition, it may predict the future consequences of changes in the number of infected at a certain period, which might be related with the flow of individuals entering or exiting a region. This study provides persuasive evidence for the predictive ability of widely disseminated social media surveys for public health applications.

## Introduction

**Figure 1:**
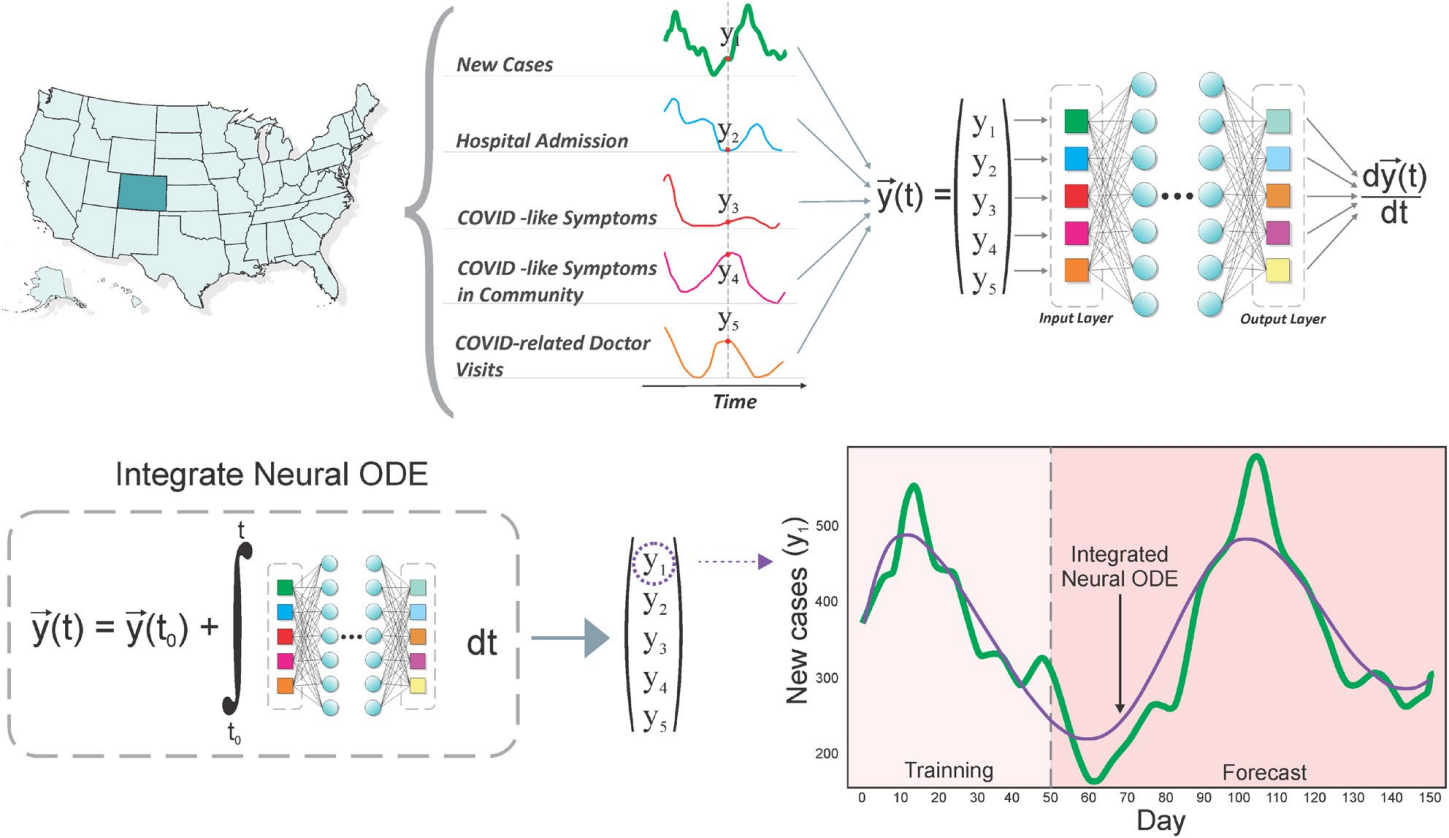
The Neural ODE is trained with a collection of signals/variables gathered from online surveys (shown above). By discovering the ordinary differential equation that best characterizes the data, the trained neural network can capture the dynamics of the temporal variation of the signals. The learned solution, derived by the temporal integration of the neural ODE, is displayed below against the reported data for newly infected cases in CO (signal $Y_1$). The solution encompasses both the training interval and the forecast.

During a pandemic, the capacity to recognize and anticipate local viral outbreaks is critical for health experts to take proper action^1,2^. However, the intrinsic parameters utilized by the prediction models to reflect the biological features of the virus cannot be determined until the pandemic has happened. While a pandemic is in progress, parameter estimation is fraught with uncertainty, which means that the first-principles models that rely on them inherit this uncertainty in their forecasts. According to one epidemiologist quoted in the New York Times^3^: “*You tell me what numbers to put in my equations, and I’ll give you the answer ...But you can’t tell me the numbers, because nobody knows them...”* , a statement that illustrates the difficulties that currently exist in predicting new infections during a pandemic.

A large amount of data is being created, either directly or indirectly, on the virus’ spread. Health Surveillance is a vital tool for forecasting, preventing, and eliminating infectious diseases and epidemics. Some of this information has been utilized for a long time in this field^4–6^. Multiple passive^4,7,8^ and active surveillance systems^9–11^ had been established across the globe^8,12^. In the last decade, new machine learning algorithms and vast data availability have enabled web-based surveillance as a supplement to conventional approaches^13–15^. Internet searches^2,16–18^, social media^19–22^, survey data^23,24^, contact tracking or monitoring using mobile devices^25,26^, and contact simulations^27^ are examples of information sources.

While digital surveillance systems offer several benefits, such as low cost, fast deployment, and extensive area coverage, they also have numerous disadvantages. For example, content and demographic bias, incomplete information, difficulty segmenting by geographic area, and complexity in digital data structure are obstacles that must be overcome^28–30^. In addition, the use of predictive models based only on big data may be hampered by lack of reproducibility, overfitting, and the necessity to continuously update algorithms (i.e., to adjust to changes in search engines)^31^. In recent years, there has been widespread agreement on the need to mix old and new information sources to enhance health surveillance model forecasts^31–33^.

The development of the COVID-19 pandemic has rekindled the hunt for more accurate forecasting models^34–36^. Large technology businesses have simultaneously made resources accessible to scholars and policymakers^37,38^. Using this information in conjunction with late indicators (such as virus positive case numbers) might be crucial to the development of more accurate outbreak prediction algorithms. The Delphi research group at Carnegie Mellon University created an API^39^ that gathers data from several sources and provides access to numerous COVID-specific and syndromic markers. This approach solves some of the aforementioned obstacles by providing simple access to geographic location and structured information. In addition, they presented a survey of symptoms developed in collaboration with universities and public health experts and presented via Facebook^40^. This social media-advertised survey reaches a wider audience and collects more information than comparable surveys. Despite the fact that the majority of web-based surveys are inexpensive and provide quick and easy access to the population, they are limited by sample bias^41^. In this case, bias was partially corrected by means of weight adjustment^40^.

It is evident from the preceding that digital information paired with first principles models or data-driven models might be used to enhance health surveillance systems. Several models have been reported to date that use various machine learning techniques and information sources^42–45^ as well as compartment modelling approaches^46–49^. In this article, we examine the predictive abilities of a state-of-the-art data-driven model to anticipate COVID-19 outbreaks using data-sets from the aforementioned symptoms survey^40^. In this case, time-dependent signals are retrieved from a particular geographic region. After processing several numerical indicators arising from the survey questions, each signal is generated. The acquired data pertains to the symptoms of individuals, infections within their social circle, hospital visits, number of internet searches for COVID-19, and average time away from home, among others. For example, a person’s input about the number of his contacts who tested positive for COVID will be linked to the number of new cases in his location.

There is no obvious model based on fundamental principles that connects the survey components to the COVID-19 numbers. However, it is plausible to predict a correlation between the local variation in time of survey answers for an area and the emergence of new viral infections in that location. In addition, these signals have the potential to serve as early indicators^44,50^, since they are not susceptible to delays caused by officially reported variables, local policies, or testing capacity.

In order to discover this relationship, a neural ordinary differential equation (neural ODE^51^) was employed to parameterize the temporal rate of change of the signal. This object employs a parameterized universal approximator to represent all conceivable phase space dynamics with a limited set of parameters that can be learnt from the training data. In this study, the neural ODE is trained on these possible early indicators and is capable of predicting viral outbreaks two months in advance. In addition, once taught, these phase space methods allow the prediction of potential future scenarios or the measurement of the uncertainty associated with changes in the number of infected in the region.

This article is divided as follows: Section “COVID-19 symptom surveys through facebook” describes the surveys and signals, as well as the arguments in favor of the notion that these signals may be utilized as early indicators. The Section “Models: first principles and data driven” provides an overview of neural ODEs and how they are used in this study. Section “Methods” describes the precise methods for incorporating the data into the neural ODEs, while “Results and discussion” section displays the predictive capability of the neural ODEs when used in this way. Finally, we examine the implications of applying these machine learning algorithms and data to health care statistics.

### COVID-19 symptom surveys through facebook

Since April 2020 universities and public health officials, in collaboration with Facebook, have been conducting a massive daily survey to monitor the spread and impact of the COVID-19 pandemic in the United States. The survey^52^ is an ongoing operation that is advertised through Facebook’s platform and is taken by nearly 55,000 people every day. Respondents provide information about COVID-related symptoms, contacts, prior medical conditions, risk factors, mental health, demographics and the economic effects of the pandemic. The information allows researchers to examine county-level trends across the US. Around 16 million responses have been collected so far.

The survey has four sections and it contains 35 questions. The first section gathers information about a set of symptoms used to define a condition called COVID-like illness (CLI), defined as fever of at least 100 $^{\circ }\hbox {F}$, along with shortness of breath, difficulty breathing or a cough^53^. Two key quantities are estimated with this information, for a given location and day:

1. The percentage of people with CLI,
2. The percentage of people who know someone in their local community with CLI illness (CLI-in-community).

The second section provides further information regarding testing, symptoms, and medical-seeking behavior. The third portion collects information on contacts and risk factors, while the fourth component collects demographic information. There is a sample of the exact questions asked in the supplemental materials. The numerical indicators (signals) are extracted from the collection of questions^52^ and responses, after a bias correction via weight adjustment^40^, resulting in a set of time series (one for each indicator) at a specified location. The aggregated data is accessible to the public via the Delphi Group websites^39,54^.

#### Surveys as early indicators

Data pertaining to the number of people who self-report CLI symptoms in a certain location may provide an early signal of COVID activity in that location. In addition, the information is not susceptible to reporting delays, unlike the formal testing metrics of confirmed daily COVID-19 cases, which are affected by testing policy and capacity.

In the Delphi Group’s^40^ Blog, it is shown that the “CLI-in-community” signal increases concurrently with confirmed COVID-19 cases, providing evidence that survey-based CLI signals can serve as early indications of COVID activity. Indeed, more individuals report that others in their neighborhood are ill when COVID-19 tests reveal an increase in confirmed cases. In fact, when COVID-19 testing indicate an increase in cases, more people report that others in their community are ill. Intriguingly, the signal begins to rise dramatically days before COVID-19 cases begin a sharp increase . This study is a non-formal examination of the indicator’s recall that permits the use of noisy and indirect signals as early indicators of new cases. Although the survey cannot be used to draw definitive conclusions about the true prevalence of coronavirus disease in the studied region, changes in self-reported symptoms over time could still be a meaningful reflection of the changes in coronavirus infections over time and could therefore assist in forecasting changes in the number of newly infected patients in the coming days.

### Models: first principles and data driven

The use of these signals for the prediction of new cases could be done by means of a model that relates the rate of variation of the different indicators to the model’s state variables. However, unlike the case of common epidemiological models, the deduction of a quantitative expression that relates the new cases as a function of the different signals extracted from the surveys is far from obvious. Even if the relations were discovered and the model was characterized, for instance, as a system of ordinary differential equations, it would undoubtedly contain unknown parameters and be subject to uncertainty. Utilizing the quantity of data and indications gathered from the surveys, a data-driven method would be a reasonable alternative for obtaining a model for forecasting new infected cases in a geographical region.

Therefore, for a particular region, we establish a vector $\vec {y}(t)$ with a sufficient collection of indicators / variables as components (including the number of new cases) and describe the model via a function that approximates the vector’s temporal evolution. With such a function, the number of new cases is expressed as a function of time, empowering prediction. In the case of the classic SIR compartment model^55^, for instance, the vector components are the variables number of susceptible individuals (S), number of infected individuals (I), and number of recovered individuals (R), along with their temporal variation expressed with an ordinary differential equation based on intuition and qualitative knowledge of the dynamics of contagions. However, in the case of the vector produced with the survey indicators as components, we cannot simply build such a model as it is not clear how to characterize the link between them from first principles.

**Figure 2:**
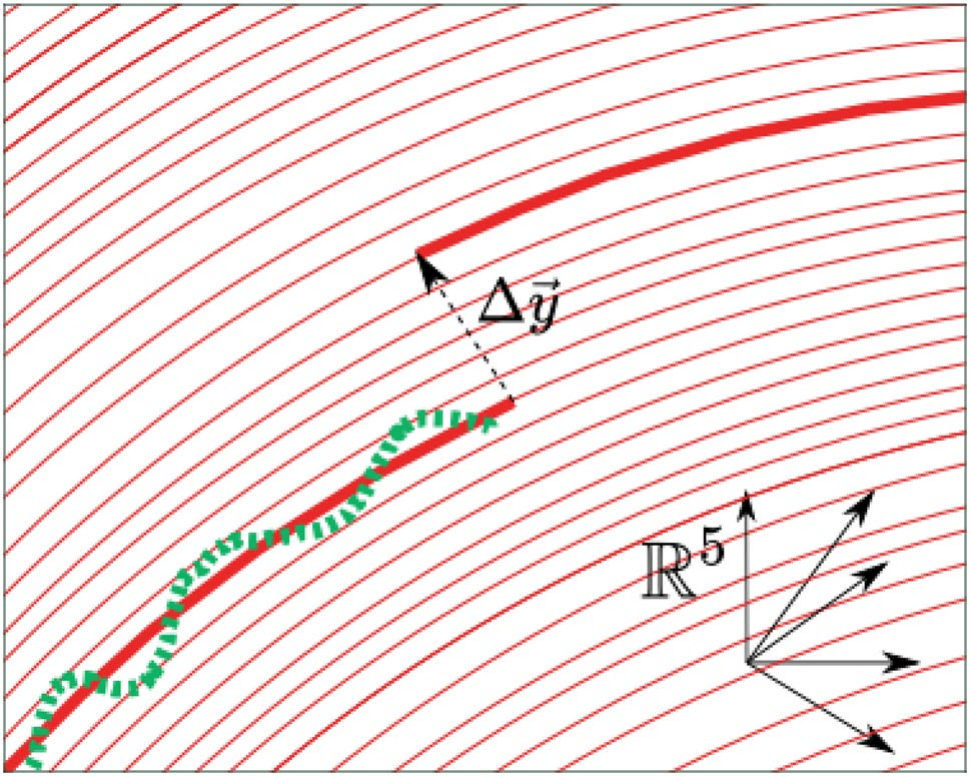
Flux line shift caused by a perturbation. If the state ${\vec {y}}(t)$ is perturbed while on a learned trajectory (thick red line), it will move to a neighboring flow line by $\Delta \vec {y}$ and continue along the solution. The green dashed line represents the five-dimensional signal extracted from the surveys and utilized to train the neural ODE. These concepts can be used to analyze disturbances in the forecast caused by abrupt changes in the number of new positive cases, which resume to changes in a single ${\vec {y}}$ coordinate.

#### Neural ordinary differential equations

Despite the absence of a known functional form that links the variables, we might examine their temporal rate of change for information. If they are represented by the vector $\vec {y}$ and it changes by $\Delta \vec {y}$ during a time interval $\Delta t$, then the rate of variation can be expressed as $\Delta \vec {y} / \Delta t$. If $\Delta t$ is sufficiently small, it may be expressed as $d\vec {y}/ d t$. Now, this expression could be approximated using a parametrized function, and if a neural network *NN* is used, it would match perfectly to the definition of a neural ordinary differential equation (neural ODE)^51^.

A Neural ODE is a neural network parametrization of an ordinary differential equation which allows for learning the dynamics of any possible dynamical system due to the universal approximation theorem^56,57^ (assuming a sufficiently large neural network). In particular, we represent our dynamical system via:

1 $$\begin{aligned} \frac{d \vec {y} }{d t} = NN(\vec {y},t,\theta ), \end{aligned}$$

where *NN* is a neural network given by weights $\theta$. This neural network has an explicit *t* dependence since it is parameterized based on the time-dependent input signals from the data. The goal is to learn the underlying dynamics of change. The “forward pass” through a neural ODE is equivalent to solving an initial value problem where $\vec {y}(t_0)$ represents the input features and a neural network substitutes hand-crafted equations. A single forward pass gives us an entire trajectory.

Unlike other architectures used for time series like residual neural networks (RNNs)^58^ or Long short-term memory (LSTMs)^59^, this model is continuous in time, allowing for incorporating non-uniform data and predictions. RNNs and LSTMs are designed for uniform time data and are equivalent to neural ODEs with uniform time steps (see^60,61^). In this sense, one could augment an RNN or LSTM with interpolations as part of the loss function; however, the accuracy of such a method for representing a continuous object is inferior to that of a differential equation solver with dense internal output (see Ref.^62^ for details). Given the constraints of the problem, it makes the most sense from a mathematical standpoint to represent the equations in this manner.

**Figure 3:**
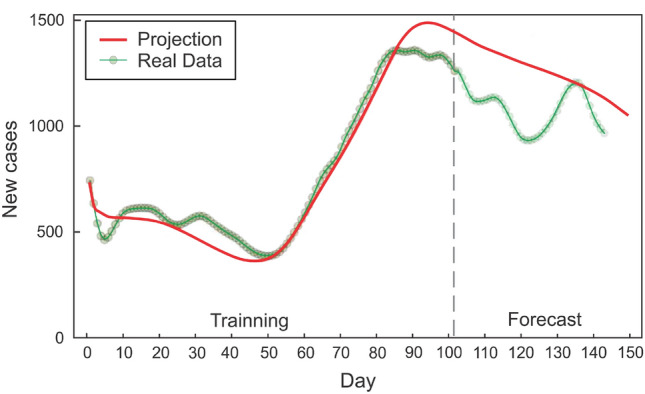
State of Ohio active case coordinate for the Neural ODE model. Newly reported cases are shown by dots, whereas the neural ODE solution is represented by a solid line. The vertical dashed line separates the training data set from the testing data set. New cases, Hospital Admission, COVID-Like Symptoms, COVID-Like Symptoms in the Community, and COVID-Related Doctor Visits are the variables included for this prediction^52^.

The parameters of the neural ODE are learned from the data as diagrammed in Fig. 1. The learning process is performed by minimizing the following loss function

2 $$\begin{aligned} L(\theta ) = \sum _{i} \left| \vec {y}(t_{i}) - \vec {y}_{data}(t_{i}) \right| ^{2}, \end{aligned}$$

with respect to the networks parameters $\theta$. Here, $\vec {y}_{data}(t_{i})$ represents the multivariate time series as a vector whose components are the values from the chosen set of signals at time $t_{i}$ while $\vec {y}(t_{i})$ is given by the numerical solution to Eq. (1). Minimization is performed by gradient-based local methods, specifically ADAM^63^. Thus in order to perform the minimization the gradient of the loss function with respect to all parameters $\theta$ must be computed. Given the large Lipshitz constants seen due to rapid changes during the onset of the growth, the adjoint technique of the original neural ODE publication is potentially unstable on the case of interest^64–66^, and thus we opted for stabilized techniques, which avoid reverse solving^67,68^.

The trained model could be applied to different initial conditions than those used during the learning procedure. With sufficient representative data, the neural ODE should be able to approximate the underlying dynamical system, particularly in the phase space region where the data was sampled. It is expected that this description will deteriorate as one moves further away. In particular, the trained neural network describes the vector field $\frac{d \vec {y} }{d t}$ in the sampled region of the n-dimensional space (n = 5 for our case. as we used 5 signals for defining the vector $\vec {y}$, see “Methods” section ). Now, if we concentrate on the initial condition ${\vec {y}}_{0}$ used for training, the integration of the neural ode from that point defines a flux line (the trajectory) , which is the same flux line that is followed to perform the extrapolation. By modifying the initial condition by a small amount, i.e. ${\vec {y}}_{0} + \Delta {\vec {y}}$, and integrating from that point, it is anticipated that the new trajectory will be similar to the previous one (see Fig. 2). As $\Delta {\vec {y}}$ increases, the flow line will move away from the training region and its error with respect to the actual trajectory (as defined by the training set) will increase. Therefore, the main assumption is that the change in the initial condition is small, and this justifies the use of the same neural network to describe the set of initial conditions close to the one used for training. The uncertainty could be estimated by measuring the sensitivity of the model, or the model fit error of the actual data for different $\Delta \vec {y}$. The method and rationale for defining the state ${\vec {y}}$ will be described in the section that follows.

**Figure 4:**
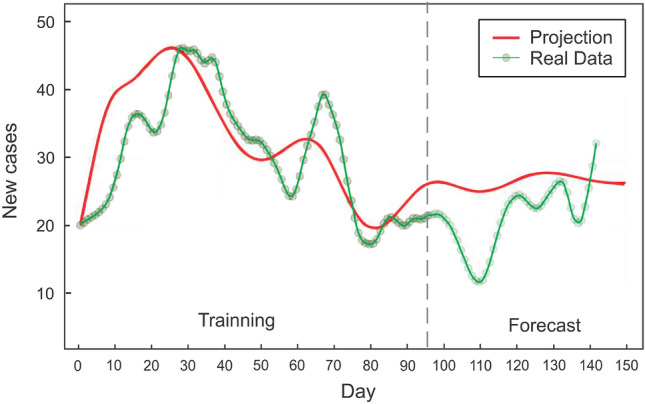
Prediction for the new infected cases in the state of ME (Maine), where the dynamics are not simply described by a model based on first principles, but the neural ODE is able to learn the dynamics and anticipate a rise in cases over the next fifty days. This rise is closely correlated with the recorded cases that were not utilized in the learning process (to the right of the vertical line).

## Methods

The raw signals for each USA State were downloaded using the Delphi Group API^39,52^. A smoothing was performed via a cubic spline interpolation for all the signals/indicators^69^ time series. The 7-day averaged of reported new confirmed COVID-19 cases was used as the main indicator of interest^70^ for accounting for the new cases. We chose the following set of variables as components in order to build the state vector *y*(*t*) for each location:

1. New daily cases (7 day averaged), (late indicator)
2. Hospital Admission, (late indicator)
3. COVID-Like Symptoms, (early indicator)
4. COVID-Like Symptoms in Community, (early indicator)
5. COVID-Related Doctor Visits (early indicator)

The collection represents an assortment of early and late correlated indicators. The complete multivariate time series is thus represented by a five-component vector ${\vec {y}}(t)$. Each coordinate represents a distinct preprocessed signal derived from surveys. One of the vector coordinates corresponds to the Active Cases signal, which is of interest. Nevertheless, the five components are utilized for learning and prediction.

The resulting multivariate time series were divided into a training set and a validation set. The training set was used to update the network’s weights $\theta$, whilst the validation set was used to assess over fitting and training generalization. Using a mini-batched^71^ variant of multiple shooting^72–74^ direct training was conducted by calculating the loss between intervals of data points. The neural ODE was solved from the point $\vec {y}_i$ at time $t_i$ to time $t_{i+1}$ with the Tsit5 method^75^ using the DifferentialEquations.jl implementation^76^ to obtain the prediction for the following point ${\vec {y}}_{i+1}$. Then, this was compared to the actual data point $\vec {y}_{data}(t_{i+1})$. The loss was computed as the mean squared error (MSE) between the observed point ${\vec {y}}_{data}(t)$ and the predicted point ${\vec {y}}(t)$ (see Eq. 2). The adjoint implementations of the DiffEqFlux.jl package^77^ were used to achieve backpropagation.

**Figure 5:**
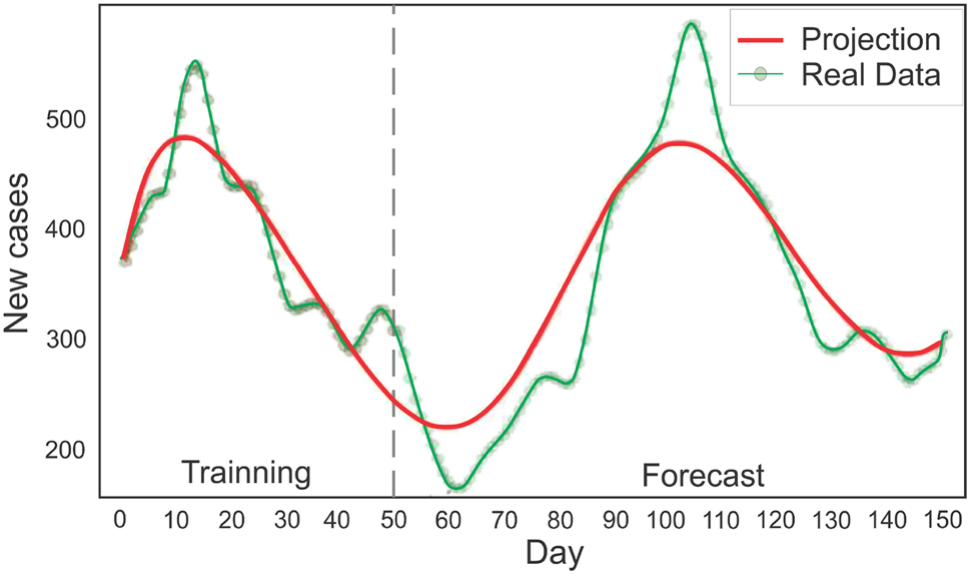
The training data set employed here (CO state) terminated before another outbreak of infected cases. Nevertheless, the neural ODE is able to determine the date of the outbreak’s peak over 60 days after its onset.

**Figure 6:**
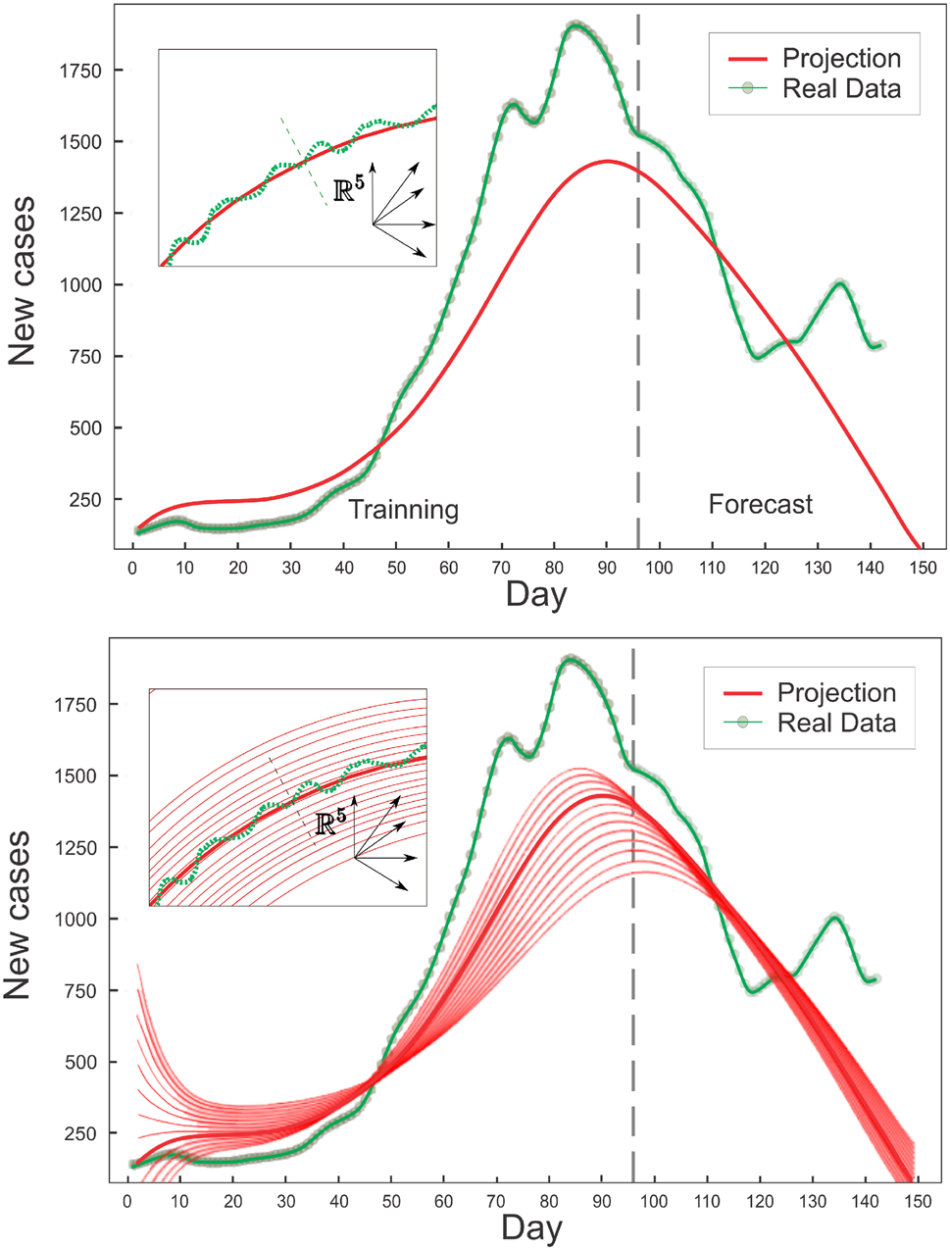
Once the neural ODE has learned the dynamics, its solution is capable of predicting the projection for a variety of initial conditions. Above, South Carolina’s new active cases (green points) and the trained neural ODE solution (thick red line) are shown. The solution is one of the five coordinates of the full vector $\vec {y}$, the trajetory of which is illustrated in the inset. By integrating the same neural ODE with different initial conditions for the number of cases, the learned phase space can be explored (illustrated below) and diverse forecasts obtained. The green represents the survey signals, whereas the thick red line represents the learned solution, as shown in the inset. The thin red lines represent phase space lines that are accessible to the system as a result of a perturbation, such as a change in the initial number of Active cases. Examining signal perturbations may help measure prediction uncertainty and estimate the effect of local active case modifications.

The neural networks used for parameterizing the ODE consisted of four interconnected layers with 64, 32, 16 and 8 neurons each and swish activation functions^78^. Once the neural net weights $\theta _i$ are calculated, the network sets the rate of the state variables’ temporal evolution (See Eq. 1). Note that this equation can be solved beyond the training interval to evaluate its forecasting accuracy. Although it is anticipated that this prediction would degrade as it goes further in phase space from the training data, we argue that a highly predictive time frame will exist.

### Software

The following open source software tools were used for this work: Pandas library^79^ for part of the data pre processing^69^, matplotlib^80^ for plotting, and Inkspace for making figures^81^, The Julia library DiffEqFlux^77,82^ for training the neural ODEs, and the differential equation library DifferentialEquations.jl^76^ for solving the differential equations.

## Results and discussion

Figure 3 displays the output and projection of the trained neural ODE for the state of Ohio. One hundred days of data were used for training. The neural ODE follows the trend of newly reported cases fifty days into the future in its forecast (see interval after the vertical dotted line). Meanwhile, Fig. 4 demonstrates the situation of the state of Maine, in which the neural ODE learns to qualitatively extrapolate the new infected cases for 40 to 50 days using data from the preceding 95 days. The dynamics of the epidemic until day 95 (the last day of training), based solely on the number of new cases, indicate a decline in contagiousness; nevertheless, the neural ODE is able to forecast an increase in the number of new cases for the subsequent fifty days. Figure 5 indicates that in the current stage, the neural ode is trained with only 50 days of data, but is able to extrapolate the epidemic dynamics for the next sixty days, predicting an increase in the number of cases 15 days after the last day of training. In addition, is able to predict the day of the next peak and subsequent decline in cases.

Once the model (the neural ODE) has learned the dynamics of the local signals, it is capable of predicting new contagions and exploring potential future scenarios in the event of signal disruptions. For instance, the trained neural ODE model might estimate the influence of a sudden shift in the number of new cases at a specific time in the future. This is due to the fact that once the neural ODE learns the dynamics of the signals, it constructs parametrically a vector field parallel to the potential trayectories of the vector ${\vec {y}}(t)$. These trajectories define flow lines contained inside the five-dimensional space. If the state ${\vec {y}}(t)$ is on a flow line and is perturbed, it will move to a neighboring flow line and continue along its route (see Fig. 2). If the perturbation is large and the state moves far from the initial flow lines specified by the training points, it is anticipated that the neural ODE will not be able to represent the vector field volume where the new flow line is located; hence, the model predictions cannot be relied upon. In this instance, however, a change in the number of active cases indicates just a disruption in one of ${\vec {y}}$’ components. If the change is moderate, the trained model should be able to characterize the trajectory of the perturbed vector without requiring the neural network to be retrained. The disruption amounts to modifying the initial condition in the neural ODE integration and may be used to analyze prediction errors brought on by signal unpredictability related to the current number of cases. Figure 6 illustrates a forecast that accounts for such uncertainty in the present epidemic data.

**Figure 7:**
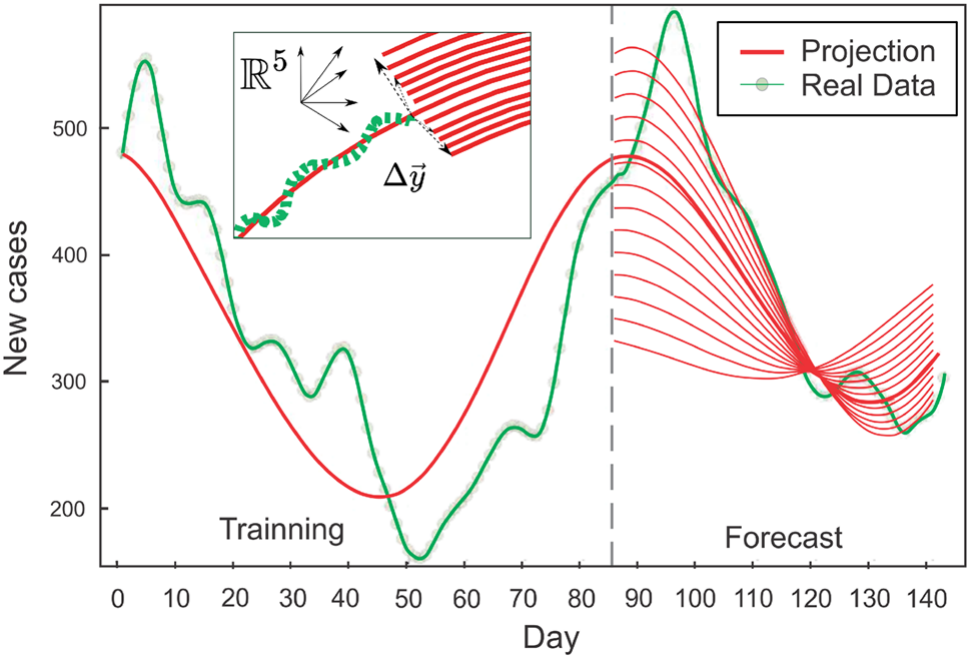
After being trained with Colorado data, the neural ODE can extrapolate new scenarios when the number of local new cases changes. This shift may occur as a result of the influx or outflow of travelers or a local immunization program. The neural ODE solution is represented by a thick line, whilst the reported 7-day average of new cases is represented by green dots. The test data begins after the vertical dashed line. Different shifts in the number of infected individuals are delineated, with the neural ode forecast for each instance displayed (thin line). The inset shows the full 5-dimensional space produced by the five survey variables and perturbation that defines possible flux lines in the system’s coordinate space. The thick red line represents the neural ODE solution, and the green line represents survey signals.

People entering or leaving the modeled area will also cause a change in the trajectory of the state vector ${\vec {y}}_0$. This is because some people will be carrying the Covid virus as they enter or leave the area. In other words, if *N* individuals visit the region, $N_{I}$ individuals will be infected, where $N_{I} \le N$. As a first approximation, we may argue that if the condition before the flow is characterized by $\vec {y}_{0}$, then the state after the flow of people is ${\vec {y}}_{0} + \Delta {\vec {y}}$, where $\Delta \vec {y} = ( N_{I}, 0, 0, 0, 0 )$ in the 5-dimensional space employed in our study. If $\Delta \vec {y}$ is not very large, we should be able to characterize the trajectory by integrating with the new beginning condition $\vec {y}_0 + \Delta \vec {y}$ (see Fig. 2). Consequently, if it is feasible to estimate the number of infected individuals among those in transit, the model might approximate the influence on the infected curve. Figure 7 showcases the varying heights of the expected peak depending on the quantity of migration chosen. By examining the perturbed solution, the change and its repercussions may be evaluated. This makes it possible for regions with strict closed borders to forecast the influence of the movement of people on the infection curve. Additionally, the impacts of immunization in the region might be measured in this manner.

The work by Mayorga et al.^83^ provides a representative example of the calculation/estimation of the infected in the flow of persons leaving or entering cities. Models of the SEIR type depict the various geographical locations and the epidemiological dynamics of their respective populations. A flow matrix that connects the local models represents the transit of individuals. On the basis of the day flow, the number of infected I and exposed E individuals is estimated and variables are updated daily. The same flow-related reasoning can be applied to our situation by substituting the SEIR models of different locations with neural ODE models trained with data extracted from local surveys.

## Conclusions and future work

Using multivariate time series connected with a geographic region, gathered by quantifying indicators from large online surveys on COVID symptoms presented via Facebook, we describe how a neural ODE can learn the dynamics that connect these variables and detect viral outbreaks in the region. We demonstrate, by analyzing data from several U.S. states, that the neural ODE is capable of forecasting up to sixty days into the future in a variety of virus-spreading scenarios.

We assert that once the neural ODE has learned the dynamics of the local signals/variables, it is capable of not only forecasting new infections in the region, but also analyzing possible future scenarios in the case of abrupt changes in the number of infected in a given day, for instance due to transit of people into or out of the analyzed region . This affords regions with strict closed borders the opportunity to predict the impact of the flow of people on the infection curve and, as a result, formulate policies in a controlled manner to optimize the transit of people and reduce economic stagnation during a pandemic.

In addition to the considerations mentioned earlier, future works can explore the potential of incorporating compression analysis into our research. While our current study focused on training the neural ODE using data from a single location or state, it is likely that the dynamics connecting local signals in one region to those in another region exhibit similar properties. Therefore, it would be valuable to investigate alternative training schemes where the model learns from multiple locations.

Furthermore, incorporating graphical models into the neural ODE framework, possibly through the utilization of graph neural networks, represents a promising avenue for future study. This approach would allow for a more comprehensive understanding of the interconnections and dependencies between different regions or entities within the system under consideration. By incorporating such graph-based techniques, we can potentially enhance the model’s predictive capabilities and capture more nuanced dynamics.

Our neural ODE model, trained on real-time social media data, extends the principles of first principles models like the SEIR model. While the SEIR model relies on assumptions about disease transmission and population dynamics, our neural ODE model directly learns from interconnected local signals extracted from social media surveys. This data-driven approach allows us to capture the underlying dynamics of disease transmission and population behavior with greater flexibility and adaptability. Moreover, our data-driven model can be combined with first principles models, such as the SEIR model, using a scientific machine learning approach^68^. By integrating the strengths of both data-driven and analytical modeling approaches, we can achieve a more comprehensive understanding of epidemic dynamics and improve the accuracy of the predictions.

This work ia a preliminary phase, a *proof of concept*. It is essential to investigate various signals and combinations and evaluate their generalization capabilities. Accurate application of the uncertainty quantification requires a great deal more research before it can be utilized in public health situations. As Nobel laureate Niels Bohr remarked a century ago: *“..prediction is very difficult, especially if it’s about the future...”*. On the other hand, these findings provide some promising outcomes for future real-time forecasts that are based on predictive data from social media.

## Supplementary Information

Supplementary Information 1.

Supplementary Information 2.

## Data Availability

All data generated or analysed during this study are included in this published article [and its supplementary information files.

## Code availability

The code supporting the current manuscript can be found in the supplementary material.
