# Supplementary material for: Forecasting virus outbreaks with social media data via neural ordinary differential equations
Source: Sci Rep. 2023 Jul 5;13:10870. doi: 10.1038/s41598-023-37118-9 (PMC10322995; doi:10.1038/s41598-023-37118-9)
Supplement: Supplementary file 1 — Supplementary Information 1. [file 41598_2023_37118_MOESM1_ESM.pdf]

Supplementay Material.

Title:

Forecasting virus outbreaks with social media data via neural ordinary differential equations

Authors:

Matías Nuñez, Nadia L. Barreiro, Rafael A. Barrio and Christopher Rackauckas

The full survey can be found in the following link: https://cmu-delphi.github.io/delphi-epidata/symptom-survey/coding.html#wave-1

The extracted signals from the survey data are updated daily and can be seen here: https://delphi.cmu.edu/covidcast/survey-results/?date=20210124

An interactive map with all the signals can be seen here https://delphi.cmu.edu/covidcast/

The following is an extract of the survey questions from which the signal where constructed.

Survey of COVID-Like Illness - US Expansion

Start of Block: Screener

You must be 18 years or older to take this survey. Are you 18 years or older?

- ○ Yes (1)
- ○ No (2)

Start of Block: Section A: Symptoms (forecast)

A1 In the past 24 hours, have **you or anyone in your household** experienced any of the following:

	Yes (1)	No (2)
Fever (100°F or higher) (1)	○	○
Sore throat (2)	○	○
Cough (3)	○	○
Shortness of breath (4)	○	○
Difficulty breathing (5)	○	○

A2 How many people in your household **(including yourself)** are **sick** (**fever**, along with **at least one other symptom** from the above list)?

A2b How many people are there in your household **in total (including yourself)**?

A3 What is your current ZIP code?

A4 How many **additional** people in your local community that you know personally are **sick** (**fever**, along with **at least one other symptom** from the above list)?

Start of Block: Section B: Symptoms (non-forecast)

B2 The rest of the survey will go into more detail to get a better understanding of your personal experience.

In the past 24 hours, have **you personally** experienced any of the following symptoms? (Select all that apply.)

- ☐ Fever (1)
- ☐ Cough (2)
- ☐ Shortness of breath (3)
- ☐ Difficulty breathing (4)
- ☐ Tiredness or exhaustion (5)
- ☐ Nasal congestion (6)
- ☐ Runny nose (7)
- ☐ Muscle or joint aches (8)
- ☐ Sore throat (9)
- ☐ Persistent pain or pressure in your chest (10)
- ☐ Nausea or vomiting (11)
- ☐ Diarrhea (12)
- ☐ Loss of smell or taste (13)
- ☐ Other (Please specify): (14) ____
- ☐ ⊗ None of the above (15)

B2b How long, in days, have you been experiencing these symptoms?

B3 You mentioned that you had a fever **in the past 24 hours**. Have you taken your temperature?

- ◯ Yes (1)
- ◯ No (2)

Q40 What was your highest temperature, in °F?

B4 You mentioned that you experienced a cough **in the past 24 hours**. Did you cough up mucus?

- ◯ Yes, I had a lot of mucus (1)
- ◯ Yes, I had a little mucus (2)
- ◯ No, I had a dry cough (3)

B5 Have you been tested for COVID-19 (coronavirus) for your current illness?

- ◯ Yes, I was tested, and received a positive diagnosis for COVID-19 (1)
- ◯ Yes, I was tested, but it was negative for COVID-19 (2)
- ◯ Yes, I was tested, but have not received the result (3)
- ◯ No, I tried to get tested but could not get a test (4)
- ◯ No, I have not tried to get tested (5)

B6 **In the past 24 hours**, have you been to the hospital to seek care for your current illness?

- ◯ Yes (1)
- ◯ No (2)
- ◯ I have tried, but been unable to receive care (3)

C1
Have you ever been told by a doctor, nurse, or other health professional that you have any of the following medical conditions?

(Please select all that apply)

- ☐ Diabetes (1)
- ☐ Cancer (other than skin cancer) (2)
- ☐ Heart disease (3)
- ☐ High blood pressure (4)
- ☐ Asthma (5)
- ☐ Chronic lung disease such as COPD or emphysema (6)
- ☐ Kidney disease (7)
- ☐ Autoimmune disorder such as rheumatoid arthritis or Crohn’s disease (8)
- ☐ ⊗None of the above (9)

C2 Have you had a flu shot in the last 12 months?

- ○ Yes (1)
- ○ No (2)

C3 **In the past 5 days**, have you gone to work outside of your home?

- ○ Yes (1)
- ○ No (2)

C4 **In the past 5 days**, have you worked or volunteered in a hospital, medical office, ambulance service, first responder services, or any other health care setting?

- Yes (1)
- No (2)

C5 **In the past 5 days**, have you worked at or visited a long-term care facility or nursing home?

- Yes (1)
- No (2)

C6 **In the past 5 days**, have you traveled outside of your state?

- Yes (1)
- No (2)

C7 To what extent are you intentionally avoiding contact with other people?

- All of the time (1)
- Most of the time; I only leave my home to buy food and other essentials (2)
- Some of the time; I have reduced the amount of times I am in public spaces, social gatherings, or at work (3)
- None of the time (4)

C8 **In the past 5 days**, how often have you ...

	None of the time (1)	Some of the time (2)	Most of the time (3)	All the time (4)
felt nervous, anxious, or on edge? (1)	○	○	○	○
felt depressed? (2)	○	○	○	○

C9 How do you feel about the possibility that you or someone in your immediate family might become seriously ill from COVID-19 (coronavirus disease)?

- Very worried (1)
- Somewhat worried (2)
- Not too worried (3)
- Not worried at all (4)

C10 **In the past 24 hours**, with how many people have you had direct contact, **outside of your household**? Your best estimate is fine.["Direct contact" means: a conversation lasting more than 5 minutes with a person who is closer than 6 feet away from you, or physical contact like hand-shaking, hugging, or kissing.]

	Number (1)
At work (1)	
Shopping for groceries and other essentials (2)	
At social gatherings (3)	
Other (4)	

C11 **In the past 24 hours**, have you had direct contact with anyone who recently tested positive for COVID-19 (coronavirus)?["Direct contact" means: a conversation lasting more than 5 minutes with a person who is closer than 6 feet away from you or physical contact like hand-shaking, hugging, or kissing.]

- ○ Yes (1)
- ○ Not to my knowledge (2)

C12 Was this person a member of your household?

- ○ Yes (1)
- ○ No (2)

A3b In which state are you currently staying?

- ○ Alabama (1)
- ○ Alaska (2)
- ○ Arizona (3)
- ○ Arkansas (4)
- ○ California (5)
- ○ Colorado (6)
- ○ Connecticut (7)
- ○ Delaware (8)
- ○ District of Columbia (9)
- ○ Florida (10)
- ○ Georgia (11)
- ○ Hawaii (12)
- ○ Idaho (13)
- ○ Illinois (14)
- ○ Indiana (15)
- ○ Iowa (16)
- ○ Kansas (17)
- ○ Kentucky (18)
- ○ Louisiana (19)
- ○ Maine (20)
- ○ Maryland (21)

- Massachusetts (22)
- Michigan (23)
- Minnesota (24)
- Mississippi (25)
- Missouri (26)
- Montana (27)
- Nebraska (28)
- Nevada (29)
- New Hampshire (30)
- New Jersey (31)
- New Mexico (32)
- New York (33)
- North Carolina (34)
- North Dakota (35)
- Ohio (36)
- Oklahoma (37)
- Oregon (38)
- Pennsylvania (39)
- Puerto Rico (40)
- Rhode Island (41)
- South Carolina (42)

○ South Dakota (43)

○ Tennessee (44)

○ Texas (45)

○ Utah (46)

○ Vermont (47)

○ Virginia (48)

○ Washington (49)

○ West Virginia (50)

○ Wisconsin (51)

○ Wyoming (52)

○ I do not reside in the United States (53)

D1 What is your gender?

○ Male (1)

○ Female (2)

○ Non-binary (3)

○ Prefer to self-describe: (4) ____

○ Prefer not to answer (5)

D1b Are you currently pregnant?

- Yes (1)
- No (2)
- Prefer not to answer (3)
- Not applicable (4)

D2 What is your age?

- 18-24 years (1)
- 25-34 years (2)
- 35-44 years (3)
- 45-54 years (4)
- 55-64 years (5)
- 65-74 years (6)
- 75 years or older (7)

D3 How many children **under 18 years old** currently stay in your household?

D4 How many adults **between 18 and 64 years old** currently stay in your household (not including yourself)?

D5 How many adults 65 years old or older currently stay in your household (not including yourself)?

Q36 How much of a threat would you say the coronavirus outbreak is to your household's finances?

- A substantial threat (1)
- A moderate threat (2)
- Not much of a threat (3)
- Not a threat at all (4)
