# Supplementary material for: Forecasting virus outbreaks with social media data via neural ordinary differential equations
Source: Sci Rep. 2023 Jul 5;13:10870. doi: 10.1038/s41598-023-37118-9 (PMC10322995; doi:10.1038/s41598-023-37118-9)
Supplement: Supplementary file 2 — Supplementary Information 2. [file 41598_2023_37118_MOESM2_ESM.pdf]

Supplementary Material

“Forecasting virus outbreaks with social media data via neural ordinary differential equations”

Matias Nuñez *et al.*

The following Julia code performs various data processing, modeling, and visualization tasks. It starts by importing necessary packages for data manipulation, machine learning, plotting, and solving ordinary differential equations. The code then loads and preprocesses data from two CSV files, extracting specific columns and applying transformations. Next, it transforms the data by rescaling it between 0 and 1 using a unit range transform. It defines a neural network model and initializes its parameters. The code includes functions for calculating loss, plotting projections, and training the model using different configurations. The training progress is visualized by plotting the loss and the model's projection. Finally, the code trains the model, iteratively updating the parameters and monitoring the loss, and plots the output of the trained model. Overall, this code combines data preprocessing, neural network modeling, and visualization to analyze and predict patterns in the given dataset.
Here's a detailed explanation of each section:

1. **Package Imports:**

   - The code starts by importing several Julia packages, including `Base.Iterators`, `Flux`, `Plots`, `Dates`, `DataFrames`, `CSV`, `OrdinaryDiffEq`, `Random`, and `DiffEqFlux`. These packages are used for data manipulation, machine learning, plotting, and solving ordinary differential equations.

```
using Statistics
using Base.Iterators: take, cycle
using Flux
using Plots
using Dates
using DataFrames, CSV
using OrdinaryDiffEq, Flux, Random
using DiffEqFlux
```

2. **Data Loading and Preprocessing:**

- Two CSV files (`_sm_oh.csv` and `_sm_co.csv`) are loaded using the `CSV.read` function and stored in the variables `data` and `data2`, respectively.
- The variable `state` is assigned the value of `data2`.
- The code then extracts various columns from `state` and assigns them to new variables using column indexing.
- Additional transformations are applied to the extracted columns, such as converting dates to numeric values and creating new columns.

```
Random.seed!(1)
data
=CSV.read("C:/cygwin64/home/matias/lab/julia/data/states_suaves/columnassinespacios/_sm_
oh.cs
v")
data2=CSV.read("C:/cygwin64/home/matias/lab/julia/data/states_suaves/columnassinespacios/
_sm_
co.csv")
state=data2
using StatsPlots

@df state plot(:time_value,[:confirmed_7dav_incidence_num])
@df state plot(:time_value,[:deaths_7dav_incidence_num])
@df state plot(:time_value,[:fbf_mas,:fbf_men])
state[:,:year] = Float64.(year.(state[:,:time_value]))
state[:,:month] = Float64.(month.(state[:,:time_value]))
state[:,:day] = Float64.(day.(state[:,:time_value]))
state[!,:date] =Float64.(1:size(state[:,:day])[1])
state[!, :cases] = state[:, :confirmed_7dav_incidence_num]
state[!, :cases_sm1] = state[:,:confirmed_7dav_incidence_num_smooth1]
state[!, :cases_sm2] = state[:,:confirmed_7dav_incidence_num_smooth2]
state[!, :dead] = state[:, :deaths_7dav_incidence_num]
state[!, :dead_sm1] = state[:, :deaths_7dav_incidence_num_smooth1]
state[!, :dead_sm2] = state[:, :deaths_7dav_incidence_num_smooth2]
state[!, :fbf] = state[:, :smoothed_hh_cmnty_cli]
state[!, :hh_sm1] = state[:,:smoothed_hh_cmnty_cli_smooth1]
state[!, :hh_sm2] = state[:,:smoothed_hh_cmnty_cli_smooth2] #community symp
state[!, :visdoc_sm2] = state[:,:smoothed_adj_cli_smooth2] #doc visits
state[!, :adj_sm2] = state[:,:smoothed_adj_covid19_smooth2] #hospital admission
state[!, :adj_sm1] = state[:,:smoothed_adj_covid19_smooth1]
state[!, :doc_sm2] = state[:,:nmf_day_doc_fbc_fbs_ght_smooth2] #combined
state[!, :fb_sm2] = state[:,:smoothed_cli_smooth2] #fb symp
```

```
state[!, :serch_sm2] = state[:,:smoothed_search_smooth2] # google
```

3. **Data Transformation:**

   - The variable `features` is defined as an array containing a subset of columns from `state`. This selection is done by specifying the desired columns in the square brackets.
   - The `unitransform` function is defined, which takes an input matrix `X` and rescales it between 0 and 1 using the `UnitRangeTransform` from the `StatsBase` package.
   - Two helper functions, `back_unitransform` and `predict_adjoint`, are also defined.
   - The code includes several commented lines that provide instructions on how to rescale and revert the rescaling of the data.

```
features = [:cases_sm2,:adj_sm2,:fb_sm2,:serch_sm2,:visdoc_sm2,:hh_sm2]
features = [:cases_sm2,:hh_sm2,:adj_sm2,:fb_sm2,:serch_sm2,:doc_sm2]
features = [:cases_sm2,:hh_sm2,:adj_sm2,:fb_sm2,:doc_sm2]
features = [:cases_sm2]
features = [:cases_sm2,:hh_sm2]
features = [:cases_sm2,:fb_sm2,:visdoc_sm2]
```

```
"""
scales X between 0 and 1. to go back use
returns rescaled X and transformation matrix dt
to go back do
 StatsBase.reconstruct(dt, copy(X))
"""
using StatsBase
function unitransform(X)
 xp = X' |> collect |> Matrix{Float64}
 dt =fit(UnitRangeTransform,xp,dims=2)
 return StatsBase.transform(dt,xp) ,dt
end
function back_unitransform(x,dt=scaleback)
 StatsBase.reconstruct(dt, copy(x))
end
function predict_adjoint(p, time_batch)
 Array(NeuralODE(f,(minimum(t), maximum(t)),Tsit5(),abstol = 1e-9,reltol =
1e9,saveat=time_batch)(y0,p))
End
```

4. **Model Training and Loss Calculation:**

- The function `loss_adjoint` is defined, which calculates the loss between the predicted and actual values given a set of parameters `p`, a batch of data `batch`, and a batch of time values `time_batch`.
- The `pl_fut` function is defined, which plots the training and test sets, as well as the projection based on the given parameters `p`.
- The code defines two arrays, `train_t` and `test_t`, containing the time values for the training and test sets, respectively.
- The arrays `train_y` and `test_y` store the corresponding target values.
- A neural network model `f` is defined using the `FastDense` layers from Flux.
- The initial parameters `p` of the neural network model are obtained using `initial_params`.
- Two functions, `minibatch` and `train`, are defined to train the model using different configurations.
- The code also includes a function `pl_ode` to plot the output of the model

```
function loss_adjoint(p, batch, time_batch)
 pred = predict_adjoint(p,time_batch)
 return sum(abs2, (batch - pred)), pred,batch
 #return Flux.msle(batch, pred), pred,batch
End

"""
plots variable var with trainning and test set plus projection
ej.
pl_fut(p,vari=1)
p NN parameters for the ODE.
w window for plotting mas menos el maximo/minimo if scale=false
vari order of the variable to be plotted in the vector features
rescale function for re escaling the variables if scale= false.
"""
function pl_fut(p, y0 = train_y[:, 1];w=400, vari = 1, scale = false,rescale=back_unitransform)
 n =Array(NeuralODE(f,(minimum(train_t), maximum(test_t)),Tsit5(),
 abstol = 1e-9,reltol = 1e-9,saveat = t[Tin:end],)(y0,p,))'
 # ymin = minimum(hcat(train_y, test_y)'[:, vari])
 # ymax = maximum(hcat(train_y, test_y)'[:, vari])
 if scale
 ymin = -0.1
 ymax = 1.4
 plot(
 n[:, vari],
 label = "Projection",
 # ylabel = features[vari],
 xlabel = "Day",
```

```
ylabel = "Cases n.u.",
xticks = 0:10:150,
linewidth = 2,
)
plot!((train_y[vari, :]), marker = 0.2, labels = "Train")
plot!(hcat(train_y, test_y)'[:, vari], marker = 0.2, labels = "Test")
plot!(
[size(train_y[1, :], 1)],
seriestype = :vline,
linecolor = :red,
line = :dash,
label = :none,
ylims = (ymin, ymax),
xticks = 0:10:150,
)
else
n2 = rescale(n')'
traine = rescale(train_y)'
test = rescale(test_y)'
ymin = minimum(hcat(traine', test')[vari, :])-w
ymax = maximum(hcat(traine', test')[vari, :])+w
plot(
n2[:, vari],
label = "Projection",
# ylabel = features[vari],
ylabel = "Cases",
xlabel = "Day",
linewidth = 2,
linecolor = :red,
xticks = 0:10:150,
)
plot!(
traine[:, vari],
marker = 0.2,
labels = "Train",
ylims = (ymin, ymax),
)
plot!(hcat(traine', test')[vari, :], marker = 0.2, labels = "Test")
plot!(
[size(train_y[1, :], 1)],
seriestype = :vline,
linecolor = :red,
line = :dash,
label = :none,
```

```
 )
 end
end
t = state[:, :date] |> t -> t .- minimum(t) |> t -> reshape(t, 1, :)
println(names(state))
plot(state.:cases_sm2)
features = [:cases_sm2,:hh_sm2,:adj_sm2,:fb_sm2,:serch_sm2,:doc_sm2]
features = [:cases_sm2,:adj_sm2,:fb_sm2,:visdoc_sm2,:hh_sm2]
features = [:cases_sm2,:adj_sm2,:fb_sm2,:visdoc_sm2,:hh_sm2]
#rescale
X = state[:, features]
res,scaleback = unitransform(Array(state[:, features]))
y = res |> y -> Matrix(y)
plot(y')
Tin = 10
Tend = 95
train_dates = state[Tin:Tend, :date]
test_dates = state[Tend+1:end, :date]
train_t, test_t = t[Tin:Tend], t[Tend:end]
train_y, test_y = y[:,Tin:Tend], y[:,Tend:end]
plot(train_t,train_y',marker = 3)
plot!(test_t,test_y')

using OrdinaryDiffEq, Flux, Random,DiffEqFlux
Random.seed!(1)
# y0=y[:, 1]
data_dim=size(y[:, 1])[1]
f = FastChain(
 FastDense(data_dim, 64, swish),
 FastDense(64, 32, swish),
 FastDense(32, 12, swish),
 FastDense(12, data_dim),
)
f = FastChain(
 FastDense(data_dim, 84, swish),
 FastDense(84, 44, swish),
 FastDense(44, 22, swish),
 FastDense(22, 12, swish),
 FastDense(12, data_dim),)
p = initial_params(f)
```

5. Model Training and Visualization:

- The code trains the model using the `train` function, specifying the learning rate (`adam`), batch size (`k`), and number of iterations (`numIter`).
- The training progress is visualized by plotting the loss and the model's projection.
- Finally, the function `pl_fut` is called to plot the output of the model.

```
using IterTools: ncycle
function minibatch(Grad = (0.001, 0.006), numbatch = (2, 3, 4, 5, 6, 7, 8,
9),numIter=150;rand=true,grafico=false)
 for adam in Grad
 for k in numbatch
 train_loader = Flux.Data.DataLoader(
 train_y,
 train_t,
 batchsize = k,
 shuffle = rand,
 )
 par = []
 losses = []
 cb = function (p, l, pred, batch; doplot = grafico) #callback function to observe training
 # println(l)
 push!(losses, l)
 push!(par, p)
 # plot current prediction against data
 if doplot
 pl = plot(pred', label = "pred", marker = 1)
 # plot(time_batch,x',marker=4)
 plot!(batch', label = "data")
 # scatter!(pl,train_t,pred[1,:],label="prediction")
 display(plot(pl))
 display(plot(losses[100:end-40]))
 end
 return false
 end
 println("minibatch= ", k)
 println("gradient step= ", adam)
 res1 = DiffEqFlux.sciml_train(
 loss_adjoint,
 p,
 ADAM(adam),
 ncycle(train_loader, numIter * 400),
 cb = cb,
 maxiters = numIter,
 )
```

```
 cb(
 res1.minimizer,
 loss_adjoint(res1.minimizer, train_y, train_t)...;
 doplot = true,
 )
 display(pl_fut(res1.minimizer))
 end
 end
end
function train(adam = 0.0006, k = 3,numIter=150;rand=false,graf=500)
 train_loader = Flux.Data.DataLoader(train_y,train_t,batchsize = k,shuffle = rand,)
 par = []
 losses = []
 cb = function (p, l, pred, batch; doplot = false) #callback function to observe training
 # println(l)
 push!(losses, l)
 push!(par, p)
 if length(losses) % graf == 0
 display(pl_fut(p))
 display(plot(losses[100:end-4]))
 end
 return false
 end
 println("minibatch= ", k)
 println("gradient step= ", adam)
 res1 = DiffEqFlux.sciml_train(loss_adjoint,p,ADAM(adam),ncycle(train_loader, numIter *
400),
 cb = cb,maxiters = numIter,)
 # cb(res1.minimizer,loss_adjoint(res1.minimizer, train_y, train_t)...;doplot = grafico,)
 display(pl_fut(res1.minimizer))
 return res1.minimizer
end
function pl_ode(p, y0 = train_y[:, 1];over=true,w=400, vari = 1, scale =
false,rescale=back_unitransform)
 n =Array(NeuralODE(f,(minimum(train_t), maximum(test_t)),Tsit5(),
 abstol = 1e-9,reltol = 1e-9,saveat=t[Tin:end])(y0,p,))'
 if over
 if scale
 plot!(
 n[:, vari],
 label = "",
 # ylabel = features[vari],
 xlabel = "Day",
 ylabel = "Infected (n.u.)",
```

```
 xticks = 0:10:150,
 linewidth = 1,
 )
 else
 n2 = rescale(n')'
 plot!(
 n2[:, vari],
 label = "",
 # ylabel = features[vari],
 ylabel = "Cases",
 xlabel = "Day",
 linewidth = 1,
 linecolor = :red,
 xticks = 0:10:150,
 )
 end
 else
 if scale
 plot(
 n[:, vari],
 label = "",
 # ylabel = features[vari],
 xlabel = "Day",
 ylabel = "Cases n.u.",
 xticks = 0:10:150,
 linewidth = 0.2,
 )
 else
 n2 = rescale(n')'
 plot(
 n2[:, vari],
 label = "",
 # ylabel = features[vari],
 ylabel = "Cases",
 xlabel = "Day",
 linewidth = 0.2,
 linecolor = :red,
 xticks = 0:10:150,
 )
 end
 end
end
p1=train(0.0003,3,3)
pl_fut(p_co;w=200, vari = 1,scale=true)
```

```
plot!(state[!, :fb_sm2])
```
